# INSTABILITY OVER THE LIFE COURSE AND POVERTY IN OLDER ADULTHOOD: A MIXED-METHODS STUDY

**DOI:** 10.1093/geroni/igz038.946

**Published:** 2019-11-08

**Authors:** Sarah Dys, Anna Steeves-Reece, Paula Carder

**Affiliations:** 1 Institute on Aging, Portland, Oregon, United States; 2 Oregon Health Science University and Portland State University School of Public Health, Portland, Oregon, United States; 3 Portland State University Institute on Aging, Portland, Oregon, United States

## Abstract

Over half of older adults (age 65+) who rent their home are cost burdened, paying more than one-third of their income on rent (JCHS). Government programs are in short supply, and most who qualify for housing assistance will wait months or years to receive a voucher or unit. This mixed methods (QUANT to qual) study explored housing instability among older adults wait-listed for housing assistance. Analysis of surveys (n=268) and in-depth interviews (n=29) examined how financial instability influences participants’ perceived risk of future homelessness. Perceived financial instability is associated with perceived risk of homelessness (p<.001). Compared to those whose current perceived financial status was now much worse from when they applied for assistance, those who were stable had lower perceived risk of homelessness. Thematic analysis of interview data provided insights into patterns of life-long experiences with various forms of instability, which are reproduced over the life course and met with little societal response or restrictive eligibility criteria. Major events (e.g., relationship changes, job loss, injury/illness) contributed to financial instability and housing instability among participants. Interviews wove together current status with individual histories, while the survey provided a cross-sectional perspective. An iterative analytic approach allowed us to conceptualize the relationships between health, employment, relationships, financial instability, food insecurity, and perceived risk of homelessness. Overall, this project describes how older Americans experience poverty and provides further evidence for how social determinants of health throughout the life course result in differential access to material resources, including income, food, health care, and housing.

